# Micro-Ultrasound: Current Role in Prostate Cancer Diagnosis and Future Possibilities

**DOI:** 10.3390/cancers15041280

**Published:** 2023-02-17

**Authors:** Adriano Basso Dias, Sangeet Ghai

**Affiliations:** Joint Department of Medical Imaging, University Health Network-Mt Sinai Hospital-Women’s College Hospital, University of Toronto, Toronto, ON M5G 2C4, Canada

**Keywords:** prostate cancer, micro-ultrasound, review, cancer detection, comparison, mpMRI

## Abstract

**Simple Summary:**

Prostate cancer (PCa) is the second most common cancer in males, and ranks second for cancer death in men. Magnetic resonance imaging (MRI) can detect PCa more accurately than biopsy alone and is now the recommended initial test in men at risk for PCa. MRI however requires additional resources and cost. Micro-ultrasound (microUS) is a new high resolution ultrasound technology that can be used to image the prostate. It integrates into the standard clinical workflow of prostate biopsy. In this review, 13 relevant publications comparing detection of clinically significant PCa between MRI and microUS were selected. The studies conclude that prostate cancer detection by microUS and MRI are comparable.

**Abstract:**

Prostate Cancer (PCa) is the second most common cancer in men. Population screening using prostate specific antigen (PSA) blood test and digital rectal exam (DRE) is recommended by the NCCN, EAU and other prominent clinical guidelines. While MRI is the recommended initial test in men at risk for PCa, micro-Ultrasound (MicroUS) is a novel high resolution ultrasound technology that has shown promise in PCa detection. This article provides a narrative review of the studies to date which have been conducted to evaluate the functionality and efficacy of MicroUS within the patient care pathway for prostate cancer. A total of 13 relevant publications comparing detection of csPCa between MicroUS and mpMRI were selected. An amount of 4 publications referring to use of MicroUS for other indications were found. Each publication was evaluated for risk of bias and applicability using the Quality Assessment of Diagnostic Accuracy (QUADAS-2) tool. The studies reviewed conclude that MicroUS detection rates for clinically significant prostate cancer diagnosis are comparable to the detection rates of mpMRI guided biopsy procedures. While the existing literature indicates that MicroUS should replace conventional TRUS for prostate imaging and biopsy, it is not yet clear whether MicroUS should be used on its own or in conjunction with mpMRI for augmenting prostate cancer detection. The ongoing OPTIMUM trial will provide evidence on how best to utilize this new technology. Early data also suggest this flexible new imaging modality has a place in local staging and active surveillance of prostate cancer as well as in bladder cancer staging.

## 1. Introduction

Prostate Cancer (PCa) is the second most common cancer in men world wide accounting for 15% of the total new cancer cases in the male population and responsible for 375,000 deaths in 2020 [1]. Population screening using prostate specific antigen (PSA) blood test and digital rectal exam (DRE) is recommended by the NCCN, EAU and other prominent clinical guidelines [2,3,4,5,6]. The PRECISION trial demonstrated that systematic biopsy is suboptimal for clinically significant prostate cancer (csPCa) detection and that the use of multiparametric Magnetic Resonance Imaging (mpMRI) targeted biopsy can increase the detection of csPCa [2,3]. This study along with others [4,5,6], have resulted in widespread adoption of mpMRI prior to biopsy and inclusion in the NCCN, EAU and other prominent clinical guidelines.

This recommendation for the use of mpMRI has revealed issues with access, as not all patients have access to high-quality mpMRI or expert radiologists. Additionally, some patients within this population will have contraindications to mpMRI, such as implanted devices, claustrophobia and impaired renal function.

**Figure 1:**
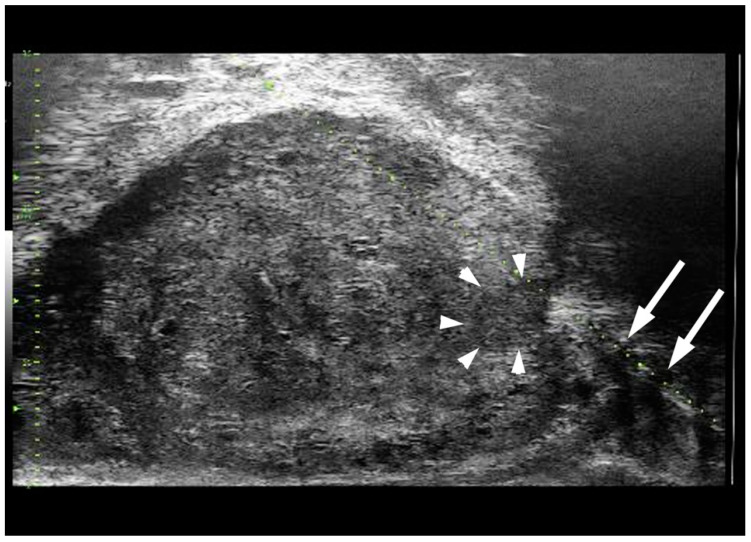
PRIMUS 4 nodule (arrowheads) identified in the apical peripheral zone on microUS with the biopsy needle (arrows). Real time visualization of the nodule allows accurate targeting without MRI/microsUS fusion.

The most recent edition of the NCCN [3], EAU [7] and AFU [8] guidelines mention the new Micro-Ultrasound (MicroUS) imaging modality for the detection of PCa citing recent studies on this topic [9,10,11]. MicroUS is a novel high resolution ultrasound technology that operates at 29 MHz. For reference, traditional urological ultrasound operates within a range of 6 to 12 MHz. This higher operating frequency allows for a spatial resolution of about 70 microns, which allows the user to visualize alterations in the ductal structure of the prostate tissue indicative for the presence of cancer [12]. Both microUS and mpMRI use a morphological assessment of the prostate, but microUS has a 10 times higher spatial resolution when compared to mpMRI. Although microUS has a higher spatial resolution, it lacks the functional imaging techniques that mpMRI can perform, this includes diffusion-weighted imaging and dynamic contrast-enhanced sequences [13]. As mpMRI has the Prostate Imaging-Reporting and Data System (PI-RADS), microUS uses the PRI-MUS (Prostate Risk Identification using Micro-UltraSound) protocol which was developed to help identify suspicious lesions of the prostate [14]. The PRI-MUS scale ranges from 1 to 5, where a lesion ranked as a 1 implies a low risk for cancer, while a lesion ranked as a 5 visualize a high risk for cancer. MicroUS paired with the PRI-MUS protocol can be used to inform biopsy decisions, guide prostate biopsy and aid in the detection of potentially cancerous regions of the prostate. Additionally, it has the ability to provide real-time visualization during the biopsy rather than requiring MRI/US fusion for targeting suspicious sites identified on mpMRI. Figure 1 shows the biopsy needle (arrows) entering a PRI-MUS 4 graded lesion (arrowheads).

This article provides a narrative review of the studies to date which have been conducted to evaluate the functionality and efficacy of MicroUS within the patient care pathway for prostate cancer.

## 2. Methods

### 2.1. Search Strategy

A narrative review was conducted examining detection rates of clinically significant cancers using MicroUS guided biopsy versus MRI guided biopsy and the use of MicroUS for other indications. Articles were found through a comprehensive search of the PubMed, Cochrane Library and Scopus databases. A list of the following keywords were used in the search: ExactVu, micro ultrasound, microUS, MUS, prostate, biopsy, 29 MHz, 29 Megahertz, Magnetic Resonance Imaging, MRI, multi-parametric, mpMRI and Fusion. The search was completed on 9 November 2022 and yielded 40 publications.

### 2.2. Inclusion and Exclusion Criteria

For this review, diagnostic accuracy studies, systematic reviews and meta-analyses strictly comparing MRI and MicroUS were selected. Studies show casing the potential use of microUS for other indications were included in this review in order to show that the uses of the technology are not fully fleshed out. Case reports and other narrative reviews were not included. Studies directly comparing MicroUS to conventional ultrasound were not included, as it is generally accepted that MicroUS is superior to conventional ultrasound. Briefly, this result has been demonstrated by Dariane et al. and Zhang et al. [15,16] and is further supported by the comparisons to mpMRI targeted biopsy since mpMRI has been demonstrated to perform better than systematic conventional ultrasound guided biopsy [2]. It is worth mentioning Zhang et al.’s findings for later comparison. Zhang et al. summarized the initial findings and experiences of early MicroUS users in a meta-analysis of 769 patients [16]. The analysis revealed that when detecting csPCa, microUS has a pooled sensitivity of 91% (95% Confidence Interval (CI) 79–97%) and pooled specificity of 49% (95% CI 30–69%) for PCa detection. This was the first meta-analysis conducted on the use of MicroUS to diagnose PCa and brought to attention the high sensitivity of this new technology.

### 2.3. Search Results and Quality Assessment

**Figure 2:**
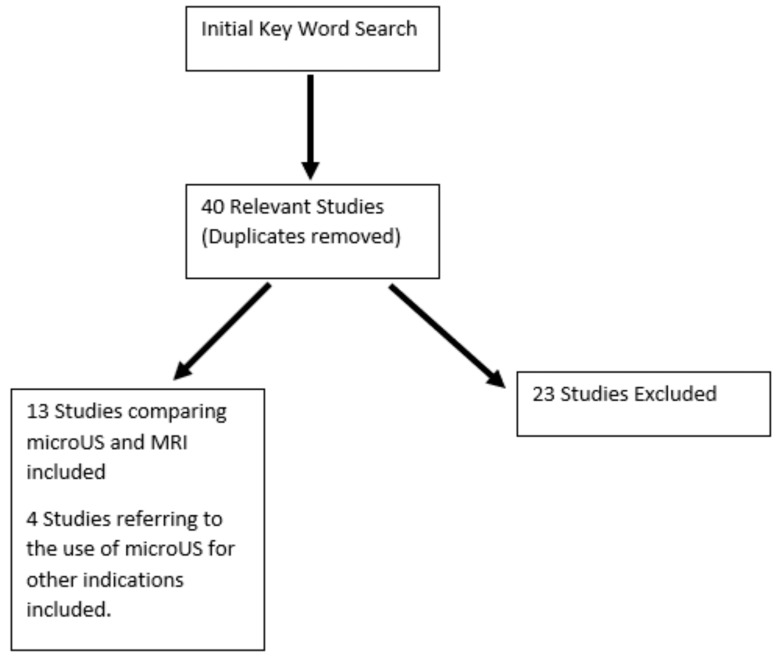
Study search and flowchart for inclusion in the review.

Of the 40 publications found only 13 relevant publications comparing detection of csPCa between MicroUS and MRI were selected for review. An amount of 4 publications referring to use of MicroUS for other indications were also found and included in this review. Figure 2 represents the study flowchart.

Each publication was evaluated for risk of bias and applicability using the Quality Assessment of Diagnostic Accuracy (QUADAS-2) tool [17], except for the meta-analyses as each article included in these analyses was assessed using the QUADAS-2 tool by the original author. This tool assesses the risk of bias and applicability of each study across 4 domains: patient selection, index testing, reference standard testing and flow and timing.

## 3. Results and Discussion

### 3.1. QUADAS-2 Results

A summary of each article’s QUADAS-2 results can be found in Supplementary Materials File. No studies showed a risk for applicability. There were four studies that showed risk of bias, three in one domain alone and one in two separate domains.

The study by Eure et al. [18], sought to compare microUS, mpMRI and conventional ultrasound when visualizing prostate cancer in nine patients in an active surveillance program. This study had a risk of bias in the reference standard as systematic biopsy guided by conventional ultrasound was used, which is known to be inferior to mpMRI targeted biopsy [2]. Having a less accurate reference standard than both index tests may make the index tests appear more effective, while not exposing the limitations of the index tests being evaluated. In this case, using systematic biopsy as the reference standard may make microUS and mpMRI seem more effective in detecting prostate cancer. The choice of reference test was justified at the time since systematic biopsy using conventional ultrasound was the standard of care.

In the study conducted by Cornud et al. [11], microUS was evaluated as a “second-look” examination to re-identify focal prostate cancer lesions and to potentially detect new lesions after undergoing bi-parametric MRI (bpMRI). The index test (microUS) was completed knowing the results of the reference standard (bpMRI) as the study attempted to discover if bpMRI lesions were visible on microUS. This introduces bias as the index test did not find lesions without assistance which could potentially over estimate the diagnostic accuracy of microUS. This is acceptable as the index test needed to be unblinded to the reference test in order to achieve the purpose of the study. This risk of bias could have been mitigated if the operator searched for lesions using microUS while blinded to the MRI.

In Lughezzani’s second study mentioned in this review [19], patients with a risk of prostate cancer as detected by mpMRI (had a lesion scored PI-RADS ≥ 3) underwent a microUS scan and biopsy to detect prostate cancer. The index test results (microUS biopsy) were not interpreted without knowing there was at least one PI-RADS ≥ 3 lesion detected using mpMRI. This would have introduced a high risk of bias as the operator would expect a lesion on the microUS machine as they target the same condition. This is acceptable as the purpose of the study was to test the diagnostic accuracy of microUS on patients suspicious for prostate cancer according to mpMRI.

The study conducted by Klotz et al. [9], was the first multi-center prospective registry trial (1040 patients at 11 sites in seven countries) to compare the sensitivity, specificity, negative predicative value (NPV) and positive predictive value (PPV) of mpMRI targeted biopsy to microUS targeted biopsy in detecting prostate. The study contains risk for bias within the patient selection criteria and the flow of the trial. This is because the inclusion criteria were designed to be broad in order to create a “real world” like registry, for example patients with prior biopsy were allowed to participate and not all patients required an mpMRI to be enrolled. This bias will inflate the detection rate of the modality used for biopsy as some patients have already been confirmed to have prostate cancer, potentially artificially increasing the detection rate of mpMRI guided biopsy or microUS guided biopsy. The issue with the flow of the trial pertains to the fact that not all patients receive the same reference standard. These issues are acceptable as the study provided one of the largest registries to date (excluding meta-analyses) with the intention of simulating the “real world” population undergoing PCa biopsy.

### 3.2. The Diagnostic Accuracy of MicroUS

The first study comparing the diagnostic accuracy of microUS to mpMRI was released in 2018 by Eure et al. [18]. This prospective study consisted of 9 patients who were on active surveillance. On the day of prostate biopsy, all patients were scanned using a conventional ultrasound followed by microUS while the operator was blinded to the mpMRI findings. Any conventional ultrasound or microUS targets identified were recorded as such. The MRI was then unblinded and fused with the real time micro-ultrasound. The clinician then targeted the areas deemed suspicious by any of the three modalities in addition to systematic 10 to 12 biopsy samples. In this initial feasibility study, the novice and expert MRI readers identified two and five targets (respectively) with Gleason Grade (GG) of 2 or above on histopathology. MicroUS identified eight lesions with GG ≥ 2. This study demonstrated that microUS could be as sensitive to csPCa as mpMRI. This feasibility study had many limitations including the small sample size, single institution and microUS reader (no inter-reader variability), lack of MRI acquisition details and lack of statistical power. The study however demonstrated that blinding between the two modalities was possible and that a within-patient comparison was feasible.

In 2019, Lughezzani et al. published the first prospective single-institutional clinical trial directly comparing microUS to mpMRI [10]. The study examined 104 patients with suspicion for PCa based on an abnormal DRE and/or an elevated PSA value while having at least one PI-RADS ≥ 3 lesion on mpMRI. The study workflow was very similar to that conducted by Eure et al. [18], except conventional ultrasound was not used and that the microUS targeting and sampling was done by one operator while the mpMRI/US targets and systematic samples were performed by a different operator allowing for improved blinding of the microUS targets and the mpMRI targets. This study identified more lesions on mpMRI (138) than microUS (117) which the authors attribute to the study design as the inclusion criteria required every patient to have one or more PI-RADS ≥ 3 mpMRI lesion. Overall, csPCa was diagnosed in 35/104 patients (34%, 95% CI 25 to 43%). Of the 35 cases of csPCa diagnosed, 2 were missed using microUS while one case was missed by both microUS and mpMRI and only found with systematic biopsy. MicroUS had a sensitivity of 94% (95% CI 81 to 98%), a specificity of 28% (95% CI 18 to 29%) and a NPV of 90% (95% CI 70 to 97). The PPV for microUS was 40% (95% CI 30 to 50%) and was 34% (95% CI 25 to 43%) for mpMRI. The authors concluded that the high sensitivity of microUS was promising, but studies with larger patient populations, and including men without mpMRI lesions as inclusion criteria, were needed.

Multiple articles highlighting the similarities of csPCa detection rates between mpMRI and microUS were published from 2019 to 2022. In 2020, Socarras et al. performed a single center prospective trial where 194 patients first underwent microUS targeted biopsy while the operator was blinded to the mpMRI results [20]. Once the microUS scan was completed, if the patient had an mpMRI lesion graded on PI-RADs as ≥ 3 they underwent MRI/US fusion targeted biopsy and systematic (saturation) biopsy. If they had no PI-RADs ≥ 3 lesions present they only underwent systematic (saturation) biopsy. MicroUS and MRI/US fusion biopsy found significantly more PCa and csPCa than systematic biopsy alone (*p* < 0.001). The investigators found no statistically significant difference in the detection rate between microUS and MRI/US (*p* = 0.15 and *p* = 0.24, respectively); however, the study was not adequately powered to identify small differences. Interestingly, microUS detected PCa in 11% (12/108 cases) of cases that were missed by the other biopsy techniques and all but one of these cases were csPCa, 10% (11/108). Systematic saturation biopsy found 4% (8/108 cases) of csPCa that were missed by microUS and MRI/US biopsy, while MRI/US biopsy found only one case of csPCa missed by microUS and systematic biopsy. This suggests that adding microUS could be more effective than mpMRI at identifying clinically significant disease and supports adding microUS targeted biopsy to existing mpMRI-based paradigms to further increase detection.

In 2021, Lugehzzani et al. performed another study to update their previously reported sensitivity and specificity of microUS with a larger cohort of 320 consecutive patients, again having at least one PI-RADS ≥ 3 lesion on mpMRI [19]. The study reported a sensitivity of 89.7% and a specificity of 26.0% for microUS. This study also found that a combination of microUS targeted and systematic biopsy found the same amount of csPCa as the combination of mpMRI targeted and systematic biopsy, suggesting that microUS has the potential to perform simillar to mpMRI targeted biopsy. In this cohort, an additional 12 cases of csPCa were missed by targeted biopsies and detected by systematic biopsy.

In 2021, Klotz et al. published the first multi-center prospective registry trial to compare the sensitivity, specificity, NPV and PPV of mpMRI targeted biopsy to microUS targeted biopsy [9]. Any man with an indication for prostate biopsy (elevated PSA or abnormal DRE) was included in the study. Prior mpMRI scans were accepted, but not required for inclusion. The procedure was similar to the other studies mentioned above, where each microUS and/or mpMRI target was sampled followed by completing standard 12–14 core systematic biopsy. The outline of the procedure was the same amongst sites, but there were methodological differences between sites. For example, 7 of the 11 sites were unblinded to the mpMRI before using microUS; however, the results between blinded and unblinded groups were similar. The study reported that for detecting csPCa (GG ≥ 2) mpMRI showed a sensitivity of 90%, a specificity of 22%, a PPV of 43% and an NPV of 77%, while microUS showed a sensitivity of 94%, a specificity of 22%, a PPV of 44% and an NPV of 85%. MicroUS was non-inferior to mpMRI for all four metrics (*p* < 0.001), and superior to mpMRI in both sensitivity and NPV (*p* < 0.03 and *p* < 0.04, respectively). However, the most important limitation was the methodological differences between sites.

Avolio et al. completed an analysis on the diagnostic accuracy of microUS in men with PI-RADS 3 lesions on mpMRI [21]. This retrospective study excluded patients with mpMRI lesions higher than PI-RADS 3 even if a PI-RADS 3 lesion was also present. The purpose was to see whether the risk stratification from microUS was useful in the case of equivocal mpMRI. The operator performed a microUS targeted biopsy while blinded to the mpMRI and then collected the mpMRI targeted samples and 6–8 core systematic biopsy. 22/111 men (20%) harbored csPCa in the cohort and at a patient level microUS demonstrated a 100% sensitivity, leading the authors to conclude that the risk stratification provided by microUS in this population may be useful. The study also reported that csPCa was detected in 4 men with PRI-MUS 3 lesions, in 15 men with PRI-MUS 4 lesions and in 3 men with PRI-MUS 5 lesions. Of the 30 patients that did not present lesions on microUS 25 (83.3%) did not have PCa while 5 (16.7%) of them were diagnosed with clinically insignificant cancer, none had csPCa. Therefore, the authors assert that these patients with PI-RADS 3 lesion on mpMRI and a negative microUS read could have avoided prostate biopsy. Among patients with at least one PRI-MUS ≥ 3 lesion, 43 (53%) had a negative biopsy, while 38 (47%) had PCa and 22 (27%) had csPCa.

In 2021, Wiemer et al. conducted a study that had operators scan and biopsy with microUS first, once completed the operator was unblinded to the MRI and took samples of the mpMRI targets if not already sampled by the microUS targets [22]. Completion systematic biopsy was performed following targeted biopsy. The study found that between the targeting capabilities of microUS and mpMRI there was no value in adding systematic biopsy. In fact, eight fewer cores per patient could have been taken while maintaining the same detection rate of csPCa. In 2022, both Ghai et al. and Hofbauer et al. published studies comparing the detection rates of csPCa with mpMRI targeted biopsy and microUS targeted biopsy [13,23]. In Hofbauer et al.’s multi-center prospective study, microUS found 73% of csPCa cases and mpMRI found 76%. MicroUS was demonstrated to be non-inferior to mpMRI (*p* = 0.023) for detecting csPCa on biopsy. In Ghai et al.’s single-center prospective trial, of the 94 men biopsied, mpMRI targeted biopsy found csPCa (GG ≥ 2) in 37 (39%) of the men and microUS found csPCa in 33 (35%) of the men. The authors conclude that the two modalities have comparable detection rates, although the study was underpowered to demonstrate non-inferiority. MicroUS would not have permitted avoidance of biopsy in as many cases as mpMRI. Promisingly, the detection of high-risk pathological features (intraductal carcinoma and cribriform subtypes) was nearly equal between modalities. The combination of the two modalities, mpMRI targeted and microUS guided biopsy, allowed the detection of csPCa in 39 (40%) of the men in the trial. This study in particular suggests the potential added benefit of using mpMRI combined with microUS and reinforced the findings by Wiemer at al that there was no value in adding systematic biopsies to mpMRI and microUS targeted biopsies.

Table 1 summarizes all of the studies reviewed that compare the detection rates of mpMRI and microUS. Studies wherein the microUS was assessed unblinded to the MRI images were not included. These 9 studies include 2234 patients over 21 institutions and provide uniform conclusions that microUS is non-inferior to mpMRI when comparing the detection rates of csPCa.

This conclusion is supported by two recent meta-analyses which compared microUS and mpMRI. The first in 2021 was completed by Sountoulides et al. [24]. A total of 13 studies were included in a qualitative analysis, which used quantitative synthesis to combine the records of 1125 patients who received microUS guided biopsy followed by mpMRI targeted and systematic biopsy. Between the three biopsy techniques 437 cases of csPCa were diagnosed. MicroUS diagnosed 341 cases while mpMRI targeted biopsy diagnosed 327 cases. These results yielded a detection ratio of 1.05 (95% CI 0.93–1.19) concluding that microUS targeted biopsy was non-inferior to mpMRI targeted biopsy.

In 2022, You et al. also conducted a meta-analysis to compare the detection rate of microUS to mpMRI targeted biopsy for PCa diagnosis [25]. The analysis included 11 studies with a total of 1081 patients. For detection of csPCa the odds ratio was calculated to be 1.01 (95% CI: 0.83 to 1.22, *p* = 0.92), meaning there was no significant difference between microUS and mpMRI in the detection of csPCa.

### 3.3. mpMRI/microUS Fusion

**Figure 3:**
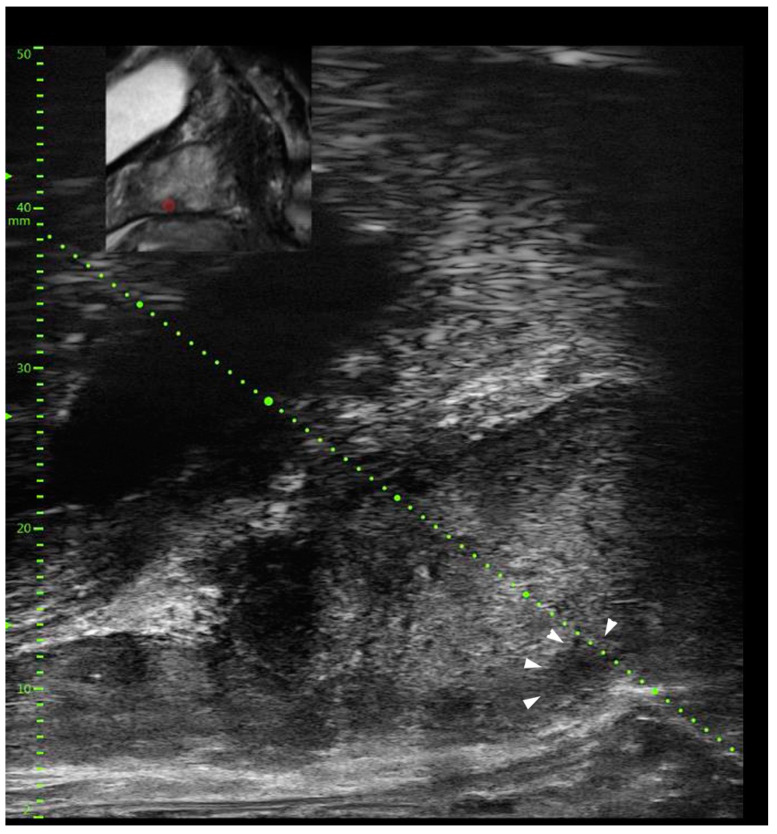
MRI lesion (red dot on MRI image in the upper left corner) is clearly visualized in real-time on microUS (outlined by the arrowheads) and provides the operator the confidence that the lesion found on MRI is the same as seen on microUS. Additionally, mpMRI/microUS fusion is beneficial in targeting MRI suspicious sites in the anterior gland, which may not be visible on microUS in real-time for biopsy.

The first commercial microUS device (ExactVu, Exact Imaging, Markham Canada) also includes the ability to perform mpMRI/microUS fusion. It has been shown that TRUS visibility of prostatic lesions facilitates targeted biopsy [26]. The ability to visualize the abnormal tissue in the real-time on microUS may therefore improve the accuracy of mpMRI-targeted biopsy by obviating the need to rely upon elastic or rigid deformation calculations and frequent corrections for patient movement or capsule marking errors (Figure 1). An example of mpMRI/microUS fusion is shown in Figure 3 wherein the MRI lesion (red dot on MRI image in the upper left corner) is clearly visualized in real-time on microUS (outlined by the arrowheads) and provides the operator the confidence that the lesion found on MRI is the same as seen on microUS. Additionally, mpMRI/microUS fusion is beneficial in targeting MRI suspicious sites in the anterior gland which may not be visible on microUS in real-time for biopsy.

The first study testing this hypothesis was conducted in 2020 by Claros et al. The study included 222 men who underwent robotic ultrasound MRI fusion biopsy and 47 men who underwent microUS targeted biopsy. The study compared the detection rate for csPCa (GG ≥ 2) between microUS/mpMRI fusion biopsy and conventional ultrasound MRI fusion biopsy [27]. The study found that microUS/mpMRI fusion biopsy detected a higher percentage of cases with clinically significant disease (38%) when compared to conventional US/MRI fusion (23%).

In 2020, Cornud et al. published a study using microUS to provide a “second-look” at lesions detection by bpMRI [11]. While the study by Cornud et al. utilized bpMRI, results are still meaningful as both bpMRi and mpMRI methods have been shown to be similar for prostate cancer detection [28]. A total of 144 lesions were detected between the two modalities. There were 114 lesions seen on both bpMRI and microUS and 13 lesions seen on only bpMRI and an additional 17 lesions visible only on microUS. CsPCa was detected in 70/114 (67.4%) of the lesions found on both microUS and bpMRI. CsPCa was not detected in any of the 13 microUS-invisible lesions found by bpMRI, while csPCa was detected in four of the bpMRI-invisible lesions seen on microUS. These results suggest that bpMRI true-positive targets are likely to be visible on microUS thereby allowing real time visualisation and targeting, rather than relying on fusion systems and the inherent concerns with this technique for targeting of MRI lesions.

Similarly, Ghai et al. [13] demonstrated that 67% of mpMRI lesions were prospectively visible on microUS allowing real-time targeting rather than requiring MRI-US fusion for targeting.

These three studies of 481 patients across three institutions all demonstrate benefits of real time targeting with the microUS device and potentially increasing csPCa detection rates when used in conjunction with mpMRI.

### 3.4. The OPTIMUM Trial

**Figure 4:**
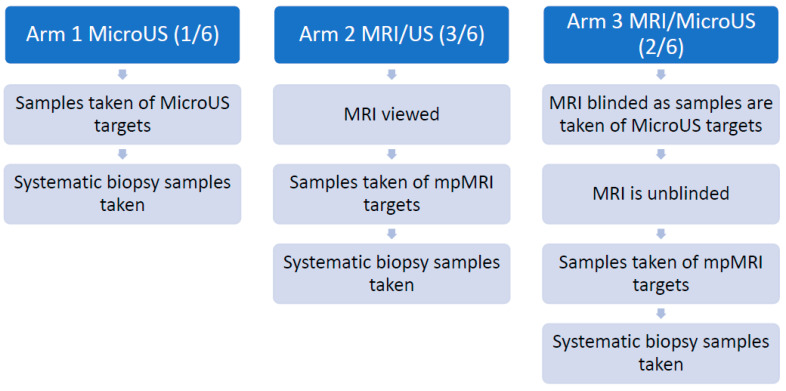
Visual representation of the OPTIMUM study workflow.

The studies reviewed conclude that microUS detection rates for csPCa diagnosis are comparable to the detection rates of mpMRI guided biopsy procedures. The conclusions presented are uniform and include three studies providing level 1a evidence (per the OCEBM criteria for diagnostics tests) [29]; however, scant randomized controlled trial data is available and some of the studies mentioned in this review have limitations with risk of bias. The OPTIMUM trial (ClinicalTrials.gov Identifier: NCT05220501), will address this unmet need. This trial proposes to enroll 1200 subjects from an expected 12 to 20 institutions. The primary outcome of the study is to compare detection of csPCa by microUS alone, and MRI/US fusion biopsy [30]. The study design includes three arms: Arm 1: microUS only biopsy, Arm 2: mpMRI/US biopsy with a conventional fusion device and Arm 3: mpMRI/microUS with microUS-based fusion. In the first two arms the relevant imaging modality (microUS or mpMRI) will guide a targeted plus systematic biopsy. The third arm of the study will function similarly to the fusion studies mentioned in this review, where the operator will take microUS targeted samples while blinded to the mpMRI, the mpMRI will then be unblinded and samples of mpMRI targets will be taken followed by a systematic biopsy. Figure 4 provides a visual representation of the study.

The 3 arms in the study will allow several secondary outcomes of interest to be calculated. These include the difference in detection of csPCa with MRI/US fusion vs. MRI/MicroUS fusion biopsy and the added value of each biopsy technique (MicroUS targeted, mpMRI targeted, and systematic). Additionally economic health data will be collected as a part of the study to assess cost and time savings compared to mpMRI targeted biopsy.

### 3.5. Other Indications of MicroUS

Given the high resolution of microUS images, the technology has been proposed to address other challenging indications within urologic oncology. These indications include local staging of PCa, active surveillance of PCa and local staging of bladder cancer.

In 2020, Regis et al. first investigated microUS’ ability to predict the presence of extraprostatic extension (EPE) before radical prostatectomy [31]. Knowledge of the existence and location of EPE allows for more confident decisions on treatment margin. In this study the patients were scanned using microUS prior to radical prostatectomy. The operators assessed for breach of the prostate capsule, irregularity or budging of the prostate capsule, loss of the prostatic-seminal vesicle angle and the presence of a hypoechoic halo and found a statistically significant relationship between the number of risk factors found on microUS and the presence of EPE. This study showed that a microUS based assessment for prediction of EPE would yield a sensitivity of 87.5% (95% CI 74.3 to 100%) and a specificity of 80.0% (95% CI 65.7 to 94.3%). The article states that MRI has a low sensitivity (55 to 61%) and a high specificity (87 to 88%) when predicting EPE [32]. This study illustrated microUS’ ability to predict non-organ-confined disease and EPE of PCa. In 2022, Fasulo et al. also investigated microUS’ ability to predict EPE of PCa prior to radical prostatectomy [33]. MicroUS correctly predicted the presence of EPE in 80% of the cases with confirmed EPE on surgery.

Active Surveillance is recommended by major urological clinical guidelines to manage patients with clinically insignificant PCa [3,7]. MicroUS’ potential role in active surveillance was recently studied by Albers et al. [34]. The study compares microUS’ ability to detect disease progression for patients within an active surveillance program from GG = 1 to GG ≥ 2, as compared to mpMRI. The study included 128 men on an active surveillance program. No difference between microUS and mpMRI was found in detecting GG ≥ 2 lesions. MicroUS (with PRI-MUS ≥ 3) had a higher sensitivity than mpMRI (with PI-RADS ≥ 3) in detecting GG ≥ 2 cancer (97% vs. 85% respectively). The study concludes that microUS may be more sensitive than mpMRI in this patient population.

Saitia et al. released an observational prospective trial to see if microUS has potential to visualize and stage bladder cancer in both males and females [35]. In the study, the microUS probe was placed transvaginally for females and transrectally for males for assessment. Of the 11 female patients there were no issues performing the scan, while 2 of the 12 males could not be scanned due to large prostate volumes that reduced the bladder window. The three layers of the bladder wall: the urothelium, the detrusor muscle and the adventitia were easily visible in healthy sections of the patient’s bladders while using microUS. MicroUS was able to reliably differentiate between non-muscle-invasive bladder cancer (NMIBC) and muscle-invasive bladder cancer (MIBC). Cancers that were larger than 5 mm were easily visualized by microUS, NMIBC appeared to bend the line of the urothelium but did not disrupt or break the continuity of that line. Conversely, MIBC appeared as tumors extending through the urothelium and into the muscular layer of the bladder. All cases identified as NMIBC on microUS were confirmed when reviewed by pathology. A total of 2 of the 7 cases identified as MIBC on microUS were downgraded to NMIBC after being reviewed by pathology.

Available evidence has demonstrated microUS to be a promising new technology with comparable cancer detection rates to mpMRI. While microUS may enable detection of some lesions not identified on mpMRI, it may have some limitations in identifying disease in the anterior transition zone specially in glands with dense corpora amylacea. Due to this, it may be best suited for use in conjunction with mpMRI, but results of the multi-center OPTIMUM trial will provide evidence for best utilization of this technology.

## 4. Conclusions

A growing body of literature is available supporting the use of MicroUS as a potentially cost and time saving technique, with comparable accuracy to mpMRI, for guidance of prostate biopsy. While the existing literature supports that MicroUS should replace conventional TRUS for prostate imaging and biopsy, it is not yet clear whether MicroUS should be used on its own or in conjunction with mpMRI for augmenting prostate cancer detection. The ongoing OPTIMUM trial will provide evidence on how best to utilize this new technology. Early data also suggest this flexible new imaging modality may also have a place in local staging and active surveillance of prostate cancer as well as in bladder cancer staging.

## Figures and Tables

**Table 1:** Summary of mpMRI and microUS comparison studies.

First Author and Reference	Year	# of Patients	Institutions	Reference Standard	Standard Biopsy Performed?	MRI Blinded or Unblinded?	MRI Parameters	MicroUS Operators	mpMRI Readers	Conclusion
Eure [18]	2018	9	1	MRI/US fusion targeted biopsy	Yes	Blinded	3.0 Tesla Toshiba Titan^TM^ no endo-rectal coil (ERC). Three-plane T2 imaging, diffusion-weighted imaging (b-value 2000) and dynamic contrast-enhanced sequences were used according to PI-RADS v2.	1	1	mpMRI found 7 lesions graded GG of 7 or above while microUS found 8.
Lughezzani [10]	2019	104	1	MRI/US fusion targeted biopsy	Yes	Blinded	Not reported	1	Not reported	MicroUS Sensitivity 94% Specificity 28%
Socarras [20]	2020	194	1	MRI/US fusion targeted biopsy (relative)	Yes	Blinded	Not reported	6	Not reported	MRI Sensitivity 84.3% Specificity 18.8% MicroUS Sensitivity 99.7% Specificity 23.1%
Lughezzani [19]	2021	320	1	MRI/US fusion targeted biopsy (relative)	Yes	Blinded	Either a 1.5 T scanner with an ERC or with a 3.0-T scanner.	2	Not reported	MicroUS Sensitivity 89.7% Specificity 26.0%
Klotz [9]	2021	1040	11	MRI/US fusion targeted biopsy (relative)	Yes	9 sites unblinded 2 sites blinded	Site A: b-value ≥ 1400 no ERC Site B: 3T Toshiba Titan no ERC b-value = 2000 Site C: 1.5T and 3T Site D: 3T Siemens Skyra no ERC Site E: 3T with pelvic phased array coil no ERC Site F: 1.5T and 3T Site G: Siemens and Phillips 3T, no ERC Site H: 1.5T and 3T Site I: 3T no ERC Site J: 1.5T and 3T Site K: 1.5T and 3T	Multiple, specific number of operators not reported	Multiple, specific number of readers not reported	MicroUS provides non-inferior Specificity and PPV and superior Sensitivity and NPV to mpMRI
Avolio [21]	2021	111	1	MRI/US fusion targeted biopsy (relative)	Yes	Blinded	1.5T scanner with an ERC or with a 3.0T scanner	2	Not reported	For MicroUS in PI- RADS 3 cases Sensitivity 100% Specificity 27.2%
Wiemer [22]	2021	159	1	MRI/US fusion targeted biopsy (relative)	Yes	Blinded	3.0T scanner with a pelvic phased-array coil without an ERC. High-spatial- resolution T2-weighted turbo spin-echo sequences in transverse and coronal orientation and transverse diffusion-weighted images (measured b values 0, 500 and 1000 s/mm^2^, calculated b value 1400 s/mm^2^) and when indicated based on the PI-RADS category of dominant sequence, a dynamic contrast-enhanced sequence.	3	Not reported	In 17% (27/159) of patients negative on mpMRI targeted biopsy were positive on microUS targeted biopsy, 7 cases were clinically insignificant prostate cancer while 20 patients had csPCa.
Hofbauer [23]	2022	203	3	MRI/US fusion targeted biopsy	Yes	Blinded	3.0T scanner with a pelvic phased-array surface coil without an ERC. T2-weighted-, diffusion-weighted-and dynamic contrast-enhanced sequences were acquired according to a PI-RADS-complaint protocol.	7	Not reported	MicroUS non-inferior to mpMRI for csPCa detection
Ghai [13]	2022	94	1	MRI/US fusion targeted biopsy (relative)	Yes	Blinded	3.0T Siemens Magnetom Skyra Fit magnet without an ERC. Sequences: T2- weighted imaging in all three planes, diffusion-weighted imaging (b values of 50, 900, 1600 s/mm^2^, with generation of apparent diffusion coefficient maps) and dynamic contrast-enhanced imaging (injection rate, mL/s).	1	1	csPCa (GG ≥ 2) rate comparable between microUS and mpMRI (35% vs. 39%)

Note—csPCa = clinically significant prostate cancer; GG = grade group; microUS = micro-ultrasound; mpMRI = multiparametric magnetic resonance imaging; PI-RADS = Prostate Imaging Reporting and Data System.

## Data Availability

The data presented in this study are available in this article (and Supplementary Material).

## Supplementary Materials

The following supporting information can be downloaded at: https://www.mdpi.com/article/10.3390/cancers15041280/s1, File S1: Summary of QUADAS-2 results of the articles assessed for the study.
