# Supplementary material for: Micro-Ultrasound: Current Role in Prostate Cancer Diagnosis and Future Possibilities
Source: Cancers (Basel). 2023 Feb 17;15(4):1280. doi: 10.3390/cancers15041280 (PMC9954149; doi:10.3390/cancers15041280)
Supplement: Supplementary file 1 [file cancers-15-01280-s001.zip › cancers-2165427-SI.pdf]

Study Name	Study Author	Domain 1 Patient Selection	Domain 2 Index Test(s)	Domain 3 Reference Standard	Domain 4 Flow and Timing	Reasoning for High/Unclear Risk or Concern	Limitations in study conclusion due to<br><br>High/Unclear Risk of Bias or Concern of Applicability
Comparison of conventional transrectal ultrasound, magnetic resonance imaging, and micro-ultrasound for visualizing prostate cancer in an active surveillance population: A feasibility study (18)	Eure et al	Risk: LOW	Risk: LOW	Risk: HIGH	Risk: LOW	Reference standard risk is high as it is unlikely to identify the target condition as recent literature has proved it is sub optimal (2).	Having a less accurate reference standard than both index tests may make the index tests appear more effective, while not exposing the limitations of the index tests being evaluated. In this case using systematic biopsy as the reference standard may make microUS and mpMRI seem more effective in detecting prostate cancer.
		Concern: LOW	Concern: LOW	Concern: LOW			
Comparison of the Diagnostic Accuracy of Micro-Ultrasound and Magnetic Resonance Imaging/Ultrasound Fusion Targeted Biopsies for the Diagnosis of Clinically Significant Prostate Cancer (10)	Lughezzani et al	Risk: LOW	Risk: LOW	Risk: LOW	Risk: LOW	None	None
		Concern: LOW	Concern: LOW	Concern: LOW			

Comparison of Initial Experience with Transrectal Magnetic Resonance Imaging Cognitive Guided Micro-Ultrasound Biopsies versus Established Transperineal Robotic Ultrasound Magnetic Resonance Imaging Fusion Biopsies for Prostate Cancer (27)	Rojas Claros et al	Risk: LOW<br>Concern: LOW	Risk: LOW<br>Concern: LOW	Risk: LOW<br>Concern: LOW	Risk: LOW	None	None
MRI-directed high-frequency (29MHz) TRUS-guided biopsies: initial results of a single-center study (11)	Cornud et al	Risk: LOW<br>Concern: LOW	Risk: HIGH<br>Concern: LOW	Risk: LOW<br>Concern: LOW	Risk: LOW	Used mpMRI to find lesions on microUS. Lesions were not found on microUS alone.	The index test was conducted with knowledge of the reference test. This introduces bias as the index test did not find lesions without assistance. It would have been better practice to search for lesions while blinded to the MRI.
Prostate Mapping for Cancer Diagnosis: The Madrid Protocol. Transperineal prostate biopsies using mpMRI fusion and micro-ultrasound guided biopsies (20)	Socarras et al	Risk: LOW<br>Concern: LOW	Risk: LOW<br>Concern: LOW	Risk: LOW<br>Concern: LOW	Risk: LOW	None	None

Diagnostic Accuracy of Micro ultrasound in Patients with a Suspicion of Prostate Cancer at Magnetic resonance Imaging: A Single-institutional Prospective Study (19)	Lughezzani et al	Risk: LOW<br>Concern: LOW	Risk: HIGH<br>Concern: LOW	Risk: LOW<br>Concern: LOW	Risk: LOW	Operators knew that PI-RADS ≥ 3 lesions were present.	The index test results (microUS biopsy) were not interpreted without knowing there was at least one PI-RADS ≥ 3 lesion present. This would have introduced a high risk of bias as the operator would expect a lesion on the microUS machine as they target the same condition.
Evolution of Targeted Prostate Biopsy by Adding Micro-Ultrasound to the Magnetic Resonance Imaging Pathway (22)	Wiemer et al	Risk: UNCLEAR<br>Concern: LOW	Risk: LOW<br>Concern: LOW	Risk: LOW<br>Concern: LOW	Risk: LOW	Patient selection is not well described, difficult to determine the risk in the patient selection process.	None
Comparison of Micro-Ultrasound and Multiparametric MRI Imaging for Prostate Cancer: Multicenter Prospective Analysis (9)	Klotz et al	Risk: HIGH<br>Concern: LOW	Risk: LOW<br>Concern: LOW	Risk: LOW<br>Concern: LOW	Risk: HIGH	Risk for patient selection bias and flow and timing was introduced as there was a broad inclusion criterion for this study.	This bias will inflate the detection rate of the modality used for biopsy as they have already been confirmed to have prostate cancer, potentially artificially increasing the detection rate of mpMRI guided

							biopsy or microUS guided biopsy.
The use of 29 MHz transrectal micro-ultrasound to stratify the prostate cancer risk in patients with PI-RADS III lesions at multiparametric MRI: A single institutional analysis (21)	Avolio et al	Risk: LOW<br>Concern: LOW	Risk: LOW<br>Concern: LOW	Risk: LOW<br>Concern: LOW	Risk: LOW	None	None
A non-inferiority comparative analysis of micro-ultrasonography and MRI-targeted biopsy in men at risk of prostate cancer (23)	Hofbauer et al	Risk: LOW<br>Concern: LOW	Risk: LOW<br>Concern: LOW	Risk: LOW<br>Concern: LOW	Risk: LOW	None	None
Optimization of prostate biopsy – Micro-Ultrasound versus MRI (OPTIMUM): A 3-arm randomized controlled trial evaluating the role of 29 MHz micro-ultrasound in guiding prostate biopsy in men with clinical suspicion of prostate cancer (30)	Klotz et al	Risk: LOW<br>Concern: LOW	Risk: LOW<br>Concern: LOW	Risk: LOW<br>Concern: LOW	Risk: UNCLEAR	Risk for Flow and Timing cannot be evaluated as the study has not been completed.	N/A

Comparison of Micro-US and Multiparametric MRI for Prostate Cancer Detection in Biopsy-Naïve men (13)	Ghai et al	Risk: LOW<br>Concern: LOW	Risk: LOW<br>Concern: LOW	Risk: LOW<br>Concern: LOW	Risk: LOW	None	None
Use of 29-MHz Micro-ultrasound for Local Staging of Prostate Cancer in Patients Scheduled for Radical Prostatectomy: A Feasibility Study (31)	Regis et al	Risk: LOW<br>Concern: UNCLEAR	Risk: LOW<br>Concern: LOW	Risk: LOW<br>Concern: LOW	Risk: LOW	Patient selection applicability concern was unclear as the patient selection criteria was not presented in great detail. Having said that all of the patients were undergoing radical prostatectomy which means they must have had prostate cancer.	None
Use of high-resolution micro-ultrasound to predict extra prostatic extension of prostate cancer prior to surgery: a prospective single-institutional study (33)	Fasulo et al	Risk: LOW<br>Concern: LOW	Risk: LOW<br>Concern: LOW	Risk: LOW<br>Concern: LOW	Risk: LOW	None	None
Micro-US vs Magnetic Resonance Imaging in	Albers et al	Risk: LOW	Risk: LOW	Risk: LOW	Risk: LOW	None	None

Prostate Cancer Active Surveillance (34)		Concern: LOW	Concern: LOW	Concern: LOW			
Assessing the Feasibility and Accuracy of High-resolution Microultrasound Imaging for Bladder Cancer Detection and Staging (35)	Saitia et al	Risk: LOW<br>Concern: LOW	Risk: HIGH<br>Concern: LOW	Risk: LOW<br>Concern: LOW	Risk: LOW	This was a feasibility study, so microUS was being used for the first time to visualize the target condition, this could have led to incorrect interpretation of the imaging.	Although the study was ranked as low for all categories of risk of bias and concern for applicability, further studies need to be conducted to determine the true feasibility of microUS for bladder cancer detection and staging.

Supplementary Materials File S1

Note: Risk: denotes the risk of bias and Concern: denotes the concern of applicability to the review question.
